# Thioacetamide-induced liver damage and thrombocytopenia is associated with induction of antiplatelet autoantibody in mice

**DOI:** 10.1038/s41598-019-53977-7

**Published:** 2019-11-25

**Authors:** You-Yen Lin, Chi-Tan Hu, Der-Shan Sun, Te-Sheng Lien, Hsin-Hou Chang

**Affiliations:** 1 Institute of Medical Sciences, Tzu-Chi University, Hualien, Taiwan, ROC; 2 Research Center for Hepatology, and Department of Gastroenterology, Buddhist Tzu Chi General Hospital, Buddhist Tzu Chi Medical Foundation, Hualien, Taiwan, ROC; 3 Department of Molecular Biology and Human Genetics, Tzu-Chi University, Hualien, Taiwan, ROC; 4 School of Medicine, Tzu-Chi University, Hualien, Taiwan, ROC

**Keywords:** Hepatitis, Innate immunity

## Abstract

Thrombocytopenia is usually associated with liver injury, elevated plasma aspartate aminotransferase and alanine aminotransferase levels, and high antiplatelet immunoglobulin (Ig) titers, although the mechanism behind these effects remains elusive. Deciphering the mechanism behind acute liver disease–associated thrombocytopenia may help solve difficulties in routine patient care, such as liver biopsy, antiviral therapy, and surgery. To determine whether liver damage is sufficient *per se* to elicit thrombocytopenia, thioacetamide (TAA)-induced hepatitis rodent models were employed. The analysis results indicated that TAA treatment transiently induced an elevation of antiplatelet antibody titer in both rats and mice. B-cell-deficient (BCD) mice, which have loss of antibody expression, exhibited markedly less thrombocytopenia and liver damage than wild-type controls. Because TAA still induces liver damage in BCD mice, this suggests that antiplatelet Ig is one of the pathogenic factors, which play exacerbating role in the acute phase of TAA-induced hepatitis. TNF-α was differentially regulated in wild-type versus BCD mice during TAA treatment, and anti-TNF treatment drastically ameliorated antiplatelet Ig induction, thrombocytopenia, and liver injury, suggesting that the TNF pathway plays a critical role in the disease progression.

## Introduction

The onset of thrombocytopenia is associated with a wide spectrum of liver diseases including viral hepatitis^1^, alcoholic hepatitis^2^, chronic hepatitis^3^, and cirrhosis^4^. The liver is the major organ expressing coagulation factors and platelet-generating hematopoietic growth factor thrombopoietin (TPO); blood clotting defects thus often occur in patients with liver diseases^1–5^. Because of an increased risk of bleeding, thrombocytopenia is a frequently observed complication in liver disease that considerably enhances the difficulty of routine patient care, such as liver biopsy, antiviral therapy, and surgery^6–9^. The suppressed production of platelets (e.g., reduced TPO expression and bone marrow suppression by hepatitis virus infection and anticancer agents) and increased consumption and destruction of platelets (e.g., splenic sequestration and coagulopathy) are generally believed to be major responses contributing to the development of thrombocytopenia in patients with liver disease^5–9^. However, another line of research has suggested that the elevation of antiplatelet (anti-PLT) immunoglobulin (Ig) may also contribute to the pathogenic progression of thrombocytopenia in liver diseases^10–13^, although the relevant mechanism remains unclear.

The inducement of anti-PLT Ig and the consequent immune thrombocytopenia (ITP; also known as immune thrombocytopenic purpura^14,15^) have been frequently observed in various forms of viral hepatitis^1–4,16^. The molecular mimicry hypothesis states that similarity between viral proteins and host antigens may cause the inducement of autoimmune Igs^17^. However, it is unclear whether the presence of autoantibodies and ITP, during the infection of four major types of hepatitis virus [hepatitis A virus (HAV), B (HBV), C (HCV), and E (HEV), which belong to the virus families *Picornavirus*, *Orthohepadnavirus*, *Hepacivirus*, and *Hepevirus*, respectively]^1,18–20^ are all attributed by molecular mimicry. In addition, the inducement of anti-PLT Ig is not exclusively observed in viral hepatitis; nonviral hepatitis, such as alcoholic hepatitis and cirrhosis, may also induce anti-PLT Ig through an unknown mechanism^2,21^, suggesting that severe liver damage, which involves acquired immune disorders, may elicit an autoimmune-prone condition.

To investigate the potential involvement of anti-PLT Ig in the exacerbation of thrombocytopenia during liver damage, plasma samples collected from hepatitis patients and thioacetamide (TAA)-treated rats and mice were analysed. We discovered that in the patients, the presence of circulating anti-PLT Ig during the acute phase was associated with high aspartate aminotransferase (AST) and alanine transaminase (ALT) levels and thrombocytopenia as compared with relatively normal platelet counts and anti-PLT Ig, AST, and ALT levels during the normal and convalescent phases. The same finding was obtained when comparing the normal, acute, and convalescent phases of TAA-treated rats and mice. The present results suggest that the inducement of anti-PLT Ig is involved in the pathogenic progression of liver-damage-associated thrombocytopenia. The relevant pathogenic progressions and effects are discussed herein.

## Materials and Methods

### Proteins and chemicals

Bovine serum albumin (BSA), apyrase, heparin, Triton X-100, ethylenediaminetetraacetic acid (EDTA), and TAA were purchased from Sigma-Aldrich (St. Louis, MO, USA). Horseradish peroxidase (HRP)-conjugated and fluorescence-labelled antibodies against human, mouse, rat, and rabbit IgG were obtained from Cappel (West Chester, PA, USA) and Jackson ImmunoResearch Laboratories (West Grove, PA, USA).

### Patients

Analyses of data and samples from human subjects were carried out in accordance with Human Subjects Research Act, Taiwan. Informed consent was obtained from all subjects; and all experimental protocols were approved by the institutional review board of Tzu-Chi Hospital (approval ID: IRB091-31). Levels of hepatitis B surface antigen (HBsAg), hepatitis B envelope antigen (HBeAg), and antibodies against HBsAg and hepatitis B core antigen HBcAg were measured using the Abbott ARCHITECT i2000SR (Abbott Laboratories, Abbott Park, North Chicago, IL, USA). HBV DNA was analysed using the analysis kit of the COBAS AmpliPrep/COBAS TaqMan HBV Test (Roche, Basel, Switzerland). Chronic HBV carriers were identified through positive detection of circulating HBsAg for more than 6 months. Reactivation of HBV was defined by more than 10-fold higher HBV DNA levels compared with the previous nadir levels or by the detection of HBeAg in the serum of patients with a negative basal circulating HBeAg level^22,23^. Acute HBV hepatitis was defined as a more than three-fold increase in circulating plasma ALT level followed by HBV reactivation. Paired blood samples collected during the normal (chronic), acute, and convalescent stages of HBV were further analysed for their platelet count and levels of plasma anti-PLT Ig, AST, and ALT. Normal blood and plasma samples were collected from sex- and age-matched adult blood donors without a history of liver disease. All plasma samples were frozen at −80 °C prior to assay.

### Analyses of AST and ALT levels

Liver damage was analysed by measuring the amounts of hepatocyte-specific enzymes AST and ALT that were released into the plasma, which was performed using previously described methods^24–26^. In brief, a blood sample from a patient or experimental animal (rats and mice) was collected and mixed with anticoagulant citrate dextrose solution (ACD; 38 mM citric acid, 75 mM sodium citrate, and 100 mM dextrose)^27,28^ in an Eppendorf tube. After centrifugation to remove the blood cells, the levels of plasma AST and ALT were then measured using a clinical biochemistry analysis system (COBAS INTEGRA 800, Roche Taiwan, Taipei, Taiwan)^24,25^ over the appropriate time courses.

### Platelet count and flow cytometry analysis of anti-PLT Ig-bound platelets

Platelet count was determined using a haematology analyser (model KX-21, Sysmex, Singapore) as described previously^29^. After incubation of a saline-diluted plasma sample (1/10; 100 μL; human, rat, or mouse) with washed platelets (1 × 10^7^; human, rat, and mouse, respectively), we determined the plasma anti-PLT Ig level through flow cytometry by detecting the platelet-bound IgG level using a fluorescence-labelled secondary antibody. Washed platelets were prepared using a previously described method^30^. To obtain washed platelets, mouse blood samples were mixed with a 1/6 volume of ACD as an anticoagulant and centrifuged at 200 × g for 15 min at room temperature. The upper platelet-rich plasma was mixed with 5 mM EDTA and recentrifuged at 1000 × g for 12 min. The supernatant was discarded, and the platelet pellet was suspended in calcium-free Tyrode’s buffer containing 0.35% BSA, heparin (50 unit/mL), and apyrase (1 unit/mL). After 20 min of incubation at 37 °C, the washed platelet pellet was resuspended in Tyrode’s buffer containing 1 mM calcium and the cell concentration was adjusted to approximately 3.5 × 108 platelets/mL. The purified platelets were then subjected to incubation and wash cycles using 1/10 diluted mouse plasma and fluorescein isothiocyanate (FITC)-labelled secondary antibody (1/2000 dilution). The relative Ig binding level on the platelets was determined using a flow cytometer (FACSCalibur; BD Biosciences, Franklin Lakes, NJ, U.S.)^14,31^. The fluorescence levels of FITC-labelled secondary antibody binding (without mouse plasma Ig binding) on platelets were considered and employed as background signals.

### Animal models and treatments

Experimental animals were maintained at a specific pathogen free condition with consistent temperature and humidity controls, and 12 h light/dark cycle^27,29,32,33^. Acute liver damage and thrombocytopenia were chemically induced using TAA in rat and mouse models by employing a method modified from those previously reported^34,35^. Eleven-week-old male Sprague–Dawley (SD) rats and 8-week-old male C57BL/6J mice weighing approximately 350–375 g and 23–25 g, respectively, were purchased from the National Laboratory Animal Center, Taiwan. The animals in the experimental groups received a single-dose (300 mg/kg for rats; 50 mg/kg for mice) intraperitoneal injection of TAA, which was dissolved in 1 × sterile saline solution (100 mg/mL), to cause acute hepatitis. Whole blood and plasma samples were collected from the experimental rats and mice at various time points before (day 0) and after (days 1–5, 7, and 10) TAA treatment (vehicle control: sterile saline solution), and the samples then underwent platelet count, enzyme-linked immunosorbent assay (ELISA), and AST and ALT analyses. Tissue section analyses were performed on tissue samples obtained from mice 2 days after treatment. To investigate the liver damage responses of TAA-treated mice without antiplatelet antibody production, B-cell-deficient (BCD) mice in C57BL/6J background (B6.129S2-*Ighm*^*tm1Cgn*^/J; backcrossed with wild-type C57BL/6J mice for more than six generations)^29^ were compared with wild-type mice. We investigated whether anti-PLT Ig could induce liver damage by immunising experimental mice (C57BL/6J) with purified rabbit (New Zealand white) platelets^14,36^ for four cycles (100 μg of platelet protein/immunisation/mouse, subcutaneously injected, with Freund’s adjuvant; Sigma-Aldrich) in 2-week intervals as described previously^37^. Recombinant glutathione *S*-transferase (GST) was purified as described^33,38,39^, and served as a control protein antigen in the aforementioned platelet-immunization experiment. Anti-PLT Ig titers were analysed using ELISA^37^. Platelet counts and plasma AST levels were determined following the methods described in previous sections. The animal study protocols complied with institutional guidelines, and were approved by the Institutional Animal Care and Use Committee (IACUC) of Tzu-Chi University (approval ID: 97066 and 103050).

### ELISA and tissue section analyses

ELISA and tissue section were employed to investigate the inflammation and liver damage of TAA-treated mice. The ELISA kit used for analysing plasma levels of proinflammatory cytokine tumour necrosis factor-α (TNF-α), high mobility group protein B1 (HMGB-1), and interleukin-6 (IL-6) levels was purchased from BioLegend (San Diego, CA, USA). An antibody used for the immunohistochemical (IHC) detection of TNF-α expression in mouse liver was obtained from R&D Systems (Minneapolis, MN, USA). Rat anti mouse IL-6 antibody was purchased from BioLegend. (San Diego, CA). Goat anti mouse HMGB1 antibody was purchased from Chondrex. (Redmond, WA). DNA staining dye 4′,6-Diamidine-2′-phenylindole dihydrochloride (DAPI) was purchased from Sigma-Aldrich. Tissue section, haematoxylin and eosin staining (H&E staining), and immunohistochemistry (IHC) were performed following previously described methods^27,40^ using an IHC kit purchased from Thermo Fisher Scientific (Fremont, CA, USA). Olympus CKX41 inverted microscope (Olympus, Tokyo, Japan) was used to capture images. The specific staining signals (e.g. green channel of TNF staining) were quantified using ImageJ software (NIH, Bethesda, MD, USA).

### Statistical analysis

The means, standard deviation (SD), and statistics of the quantifiable data were calculated using Microsoft Office Excel 2003, SigmaPlot 10, and SPSS 17, respectively. The significance of the data was examined using one-way analysis of variance (ANOVA), followed by the post hoc Bonferroni-corrected *t* test. A probability of type 1 error (α = 0.05) was recognized as the threshold for statistical significance.

## Results

### Reduced anti-PLT Ig level is associated with ameliorated thrombocytopenia and AST and ALT levels during the convalescent phase

**Figure 1:**
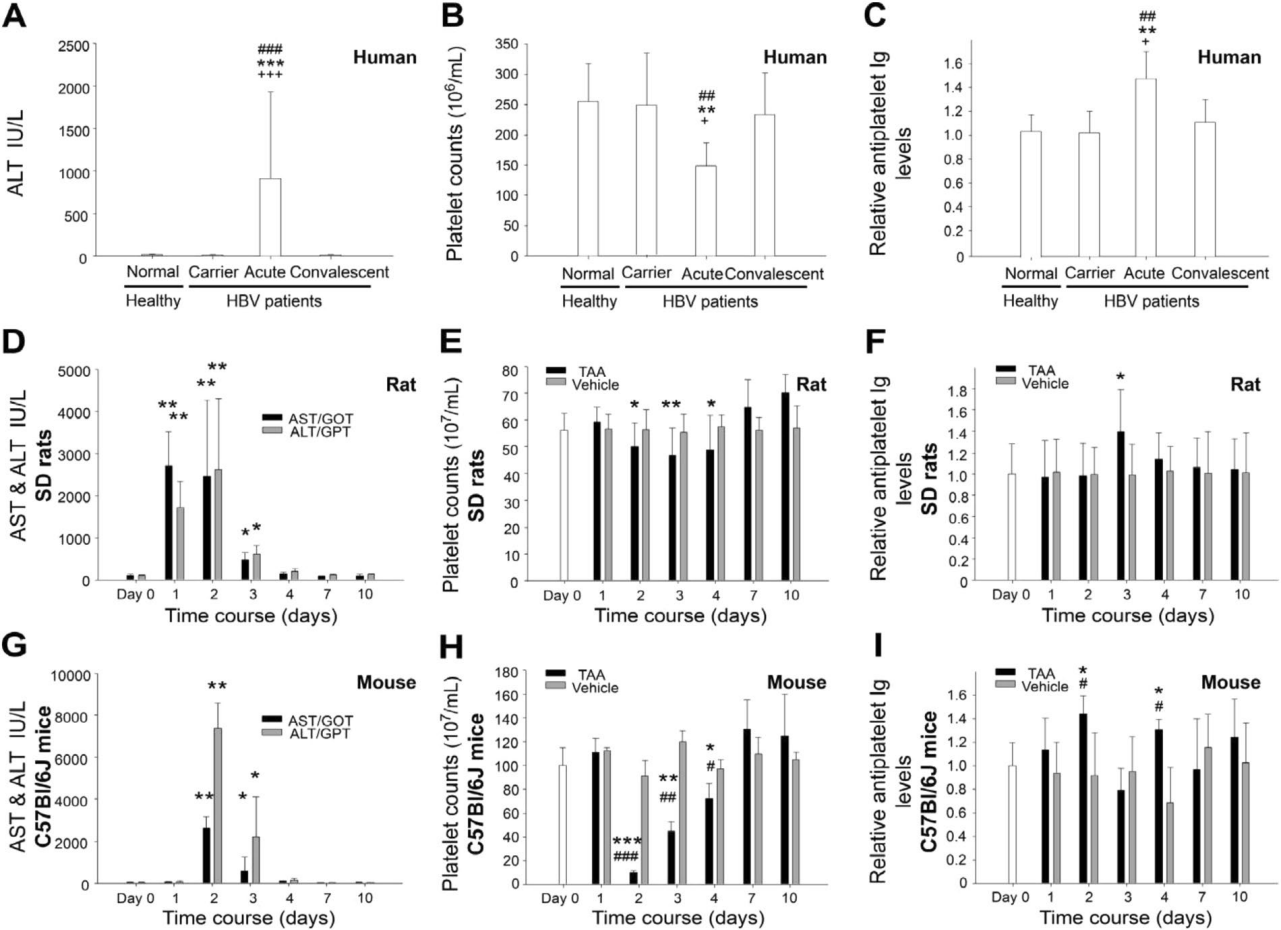
Acute liver damage associated with induction of antiplatelet immunoglobulin and thrombocytopenia. Plasma ALT (**A**,**D**,**G**) and AST levels (**D**,**G**) platelet counts (**B**,**E**,**H**) and antiplatelet immunoglobulin (anti-PLT Ig; **C**,**F**,**I**; “normal” group in **C**, and Day 0 groups in **F**,**I** were normalized to 1 fold) and in HBV patients, TAA treated rats (**D**–**F**) and mice (**G**–**I**). The “normal” indicated a stable stage of chronic hepatitis B virus-infected patient without obvious hepatic injury; the “acute” indicated a stage with recurrent hepatitis and viral activities (**A**–**C**). Normal healthy control n = 6; HBV patients n = 5 (**A**–**C**), n = 18 (**D**–**F**), n = 6 (**G**–**I**). ^##^*P* < 0.01, ^###^*P* < 0.001, (**A**–**C**) vs. normal healthy controls; **P < 0.01, **P < 0.01, ***P < 0.001, (**A**–**C**) vs carrier state; ^+^*P* < 0.01, ^+++^*P* < 0.001, (**A**–**C**) vs convalescent state, *P < 0.05, **P < 0.01, ***P < 0.001, (**D**–**I**) vs. respective day 0 groups; ^#^*P* < 0.05, ^##^*P* < 0.01, ^###^*P* < 0.001, (**D**–**I**) vs. respective vehicle groups.

Autoimmunity is one of the pathogenic mechanisms that induces liver damage in patients with viral hepatitis^41,42^. Using paired blood samples from patients with HBV, we analysed the presence of anti-PLT Ig and thrombocytopenia in different liver damage progression stages (carrier state, acute, and convalescent). We discovered that the presence of anti-PLT Ig is associated with thrombocytopenia, specifically during the acute phase (Fig. 1A–C, normal and carrier vs. acute, ^##^*P* < 0.01, ^###^*P* < 0.001, ^**^P < 0.01, ^***^P < 0.001), but the anti-PLT Ig level and platelet count returns to normal in the later convalescent phase (Fig. 1A–C, acute vs. convalescent, ^+^*P* < 0.05, ^+++^*P* < 0.001). Our data suggested that the inducement of anti-PLT Ig is associated with liver damage and thrombocytopenia in the acute phase of viral hepatitis.

**Figure 2:**
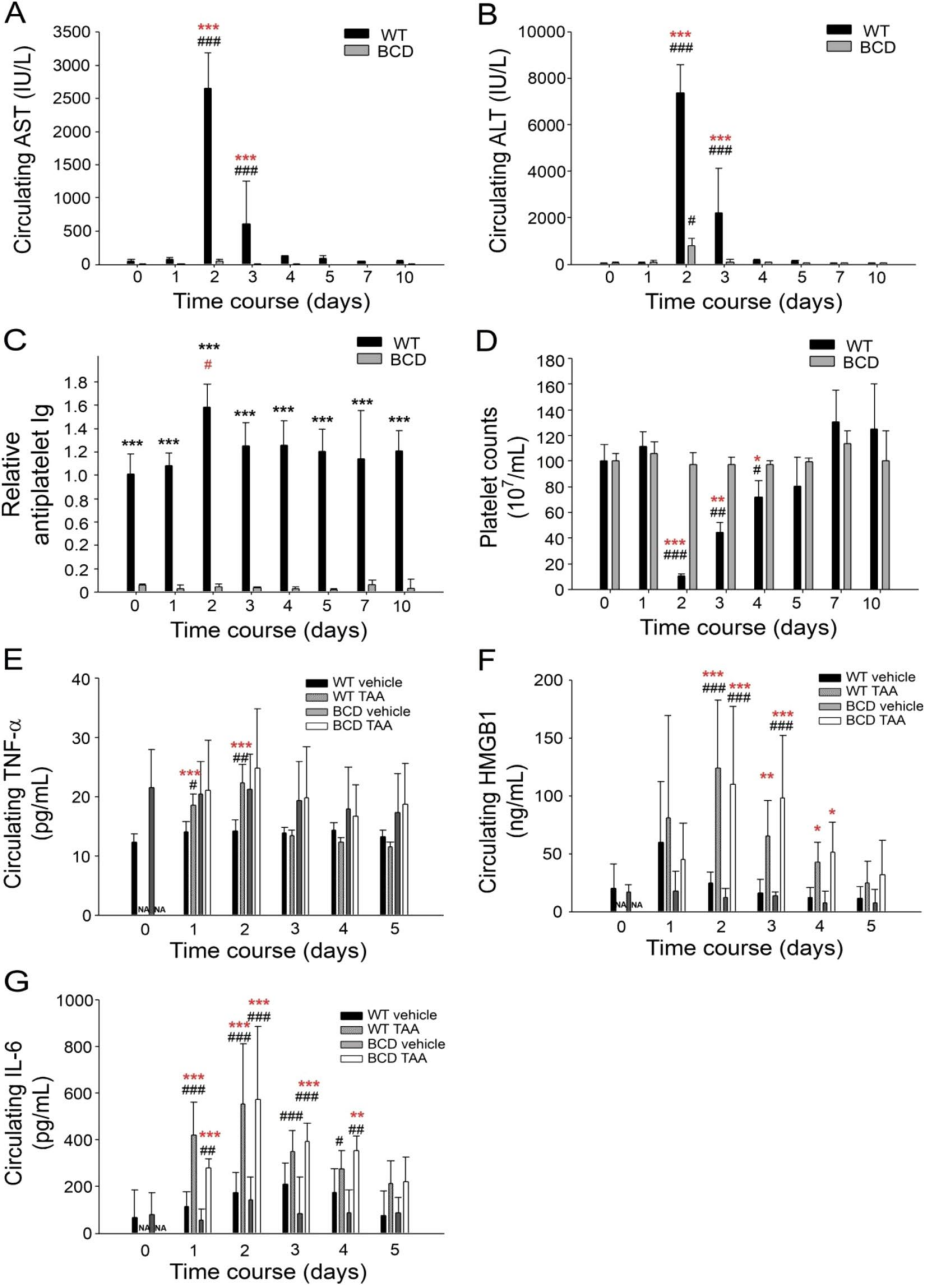
B cell deficient (BCD) mice displayed markedly less liver damage, anti-PLT Ig, thrombocytopenia and TNF expression versus wild type mice. TAA-mediated induction of circulating AST (**A**), ALT (**B**), anti-PLT Ig (**C**; WT Day 0 groups were normalized to 1 fold), PLT counts (**D**), TNF-α (**E**), HMGB1 (**F**), and IL-6 (**G**) levels in B cell deficient (BCD) vs. wild type (WT) mice were shown. n = 6, ^#^*P* < 0.05, ^##^*P* < 0.01, ^###^*P* < 0.001 vs. respective day 0 groups; vs. ^*^P < 0.05, ^**^P < 0.01, ^***^P < 0.001 WT vs. BCD (**A**–**D**), vs. respective vehicle groups (**E**–**G**).

Animal models of acute liver injury caused by hepatotoxic chemical TAA treatment were employed to further investigate whether liver damage without the presence of a foreign viral antigen is sufficient to elicit anti-PLT Ig. Intriguingly, we discovered that TAA-induced liver damage (increased AST and ALT levels; Fig. 1D,G; day 1–3 vs. day 0, *P < 0.05, **P < 0.01, ***P < 0.001; TAA vs. vehicle, ^#^*P* < 0.05, ^##^*P* < 0.01, ^###^*P* < 0.001) was associated with the induction of thrombocytopenia (Fig. 1E,H, day 1–3 vs. day 0, *P < 0.05, **P < 0.01, ***P < 0.001; TAA vs. vehicle, ^#^*P* < 0.05, ^##^*P* < 0.01, ^###^*P* < 0.001) and relatively higher anti-PLT Ig levels (Fig. 1F,I, *P < 0.05 vs. day 0; ^#^*P* < 0.05, TAA vs. vehicle) in both rat (Fig. 1D–F) and mouse (Figs. 1G–I and 2A,B) models. Anti-PLT Ig was elicited within 2 days of TAA treatment (Figs. 1F,I and 2C), suggesting that this response was not a conventional adaptive immune response. Despite the total circulating IgG levels were not markedly changed during liver damage in human subjects, rats, and mice; mouse plasma IgG levels tended to be up-regulated during liver damage (Fig. S1). Because strong inflammation was induced (please see the following sections), this was likely due to excess-inflammation-triggered abnormal B cell activation, as described elsewhere^43–46^; why the autoreactive Ig targeted the platelets, however, is unclear and worthy of further investigation.

### TAA cannot induce anti-PLT Ig and strong liver damage in BCD mice

According to the results presented in the previous section, if anti-PLT Ig is indeed involved in the induction of thrombocytopenia, mice deficient in Ig production should exhibit lower thrombocytopenic responses after TAA treatment. Knockout mice deficient in the constant region of the immunoglobulin heavy chain gene (*Ighm*^−/−^; C57BL/6J), mice that cannot produce mature B cells and have plasma-Ig-deficient and BCD phenotypes^29^, were employed to investigate the role of antibody expression in TAA-induced thrombocytopenia and liver damage. Intriguingly, when compared with the wild-type controls, which exhibited strong platelet count reduction and severe liver injury, the BCD mice developed markedly less liver damage (Fig. 2A,B), and thrombocytopenia (Fig. 2D; ^#^*P* < 0.05, ^##^*P* < 0.01, ^###^*P* < 0.00, day 2 vs. day 0; *P < 0.05, **P < 0.01, ***P < 0.001 WT vs. BCD) in association with their loss of production of anti-PLT Ig (Fig. 2C, ^#^*P* < 0.05, vs. day 0) after TAA treatment; and can be showed as a dose dependent response (Figs. S2 and S3). Next, we analysed the proinflammatory cytokine TNF-α, HMGB-1, and IL-6 levels of TAA-treated wild-type versus BCD mice; theoretically, the cytokine differently regulated in wild-type versus BCD mice is associated with the aforementioned abnormal anti-PLT Ig induction. The TNF-α data immediately attracted our attention because the plasma TNF-α levels were markedly increased only in wild-type but not BCD mice by Days 1 and 2 after TAA treatment (Fig. 2E, ^***^P < 0.001, day 1, day 2, WT TAA vs. WT vehicle). Conversely, the HMGB1 and IL-6 levels were increased in both the wild-type and BCD mice by Day 2 after TAA treatment (Fig. 2F,G, significantly up-regulated vs. 2E, not significantly up-regulated, BCD TAA vs. BCD vehicle groups). Because BCD mice do not express anti-PLT Ig, this suggests that HMGB1 and IL-6 may be not essential to TAA-induced liver damage and anti-PLT Ig in wild-type mice.

### TNF-α plays critical role in TAA-induced liver damage

**Figure 3:**
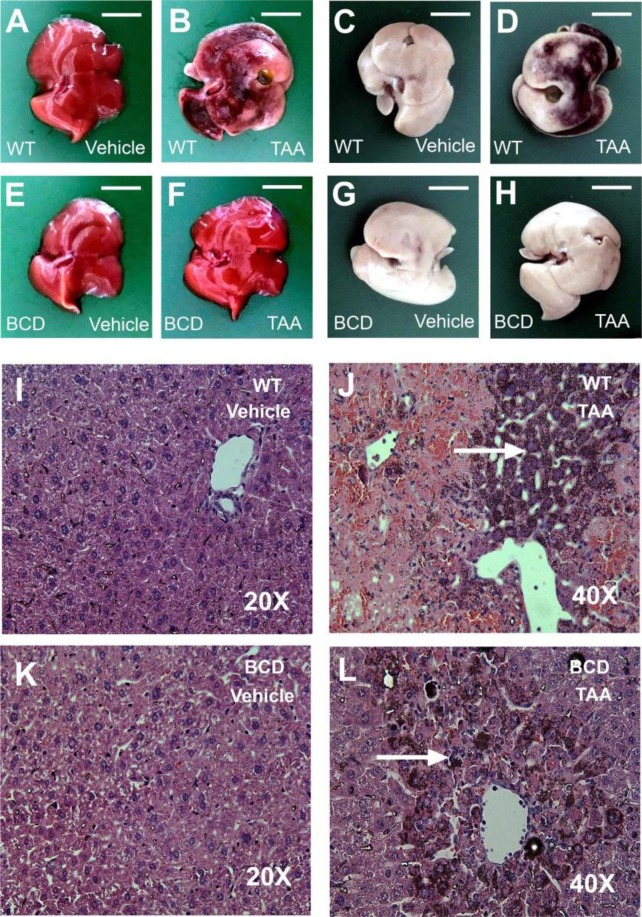
Liver and hematoxylin-eosin (H&E) stain of mouse liver. Images of liver collected from mouse treated with or without TAA before and after formalin fixation are showed (**A**–**H**; scale bar 1 cm). H&E staining of liver section from mouse treated with (**I**,**K**) or without TAA (**J**,**L**). Arrow indicated areas are TAA-induced necrotic lesions (**J**,**L**).

**Figure 4:**
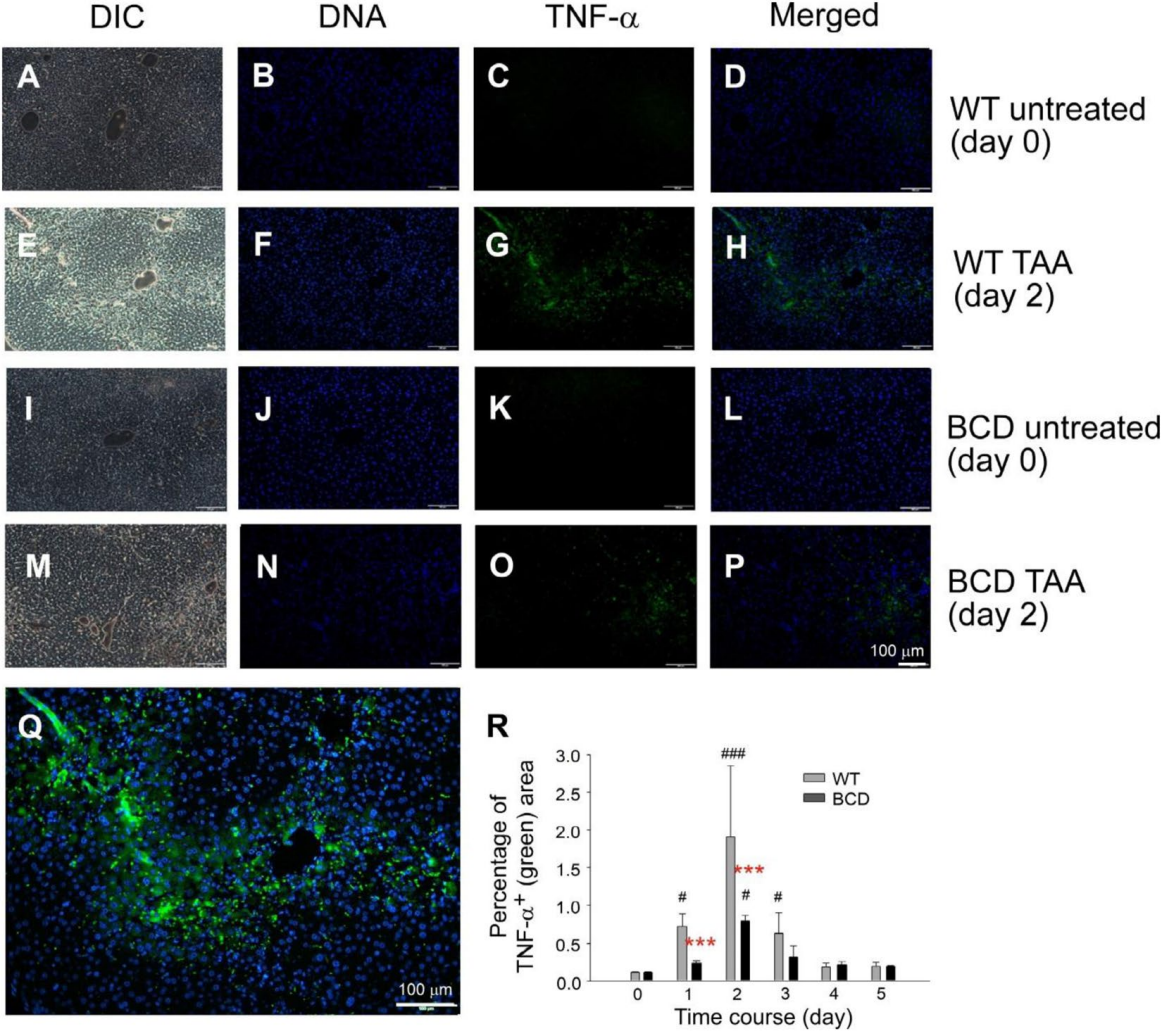
TNF-α immunohistochemistry (IHC) of mouse liver. Liver samples of wild type (WT; **A**–**H**,**Q**) and B cell deficient (BCD; **I**–**P**) mice (in C57BL/6J background) treated with TAA (WT, **E**–**H**,**Q**; BCD, **M**–**P**), or without (untreated day 0; WT, **A**–**D**; BCD, **I**–**L**) were analyzed by IHC to detect the TNF-α expression. Representative images are showed. To highlight the image details of TNF-α staining, Q is showed as an enlarged image of H. DIC: bright field of differential interference contrast image; DNA: stained with DAPI. ImageJ quantified TNF-staining signals are showed in R. ^#^*P* < 0.05, ^###^*P* < 0.001 vs. respective day 0 groups; ***P < 0.001 vs. respective WT groups. Scale bars 100 μm.

**Figure 5:**
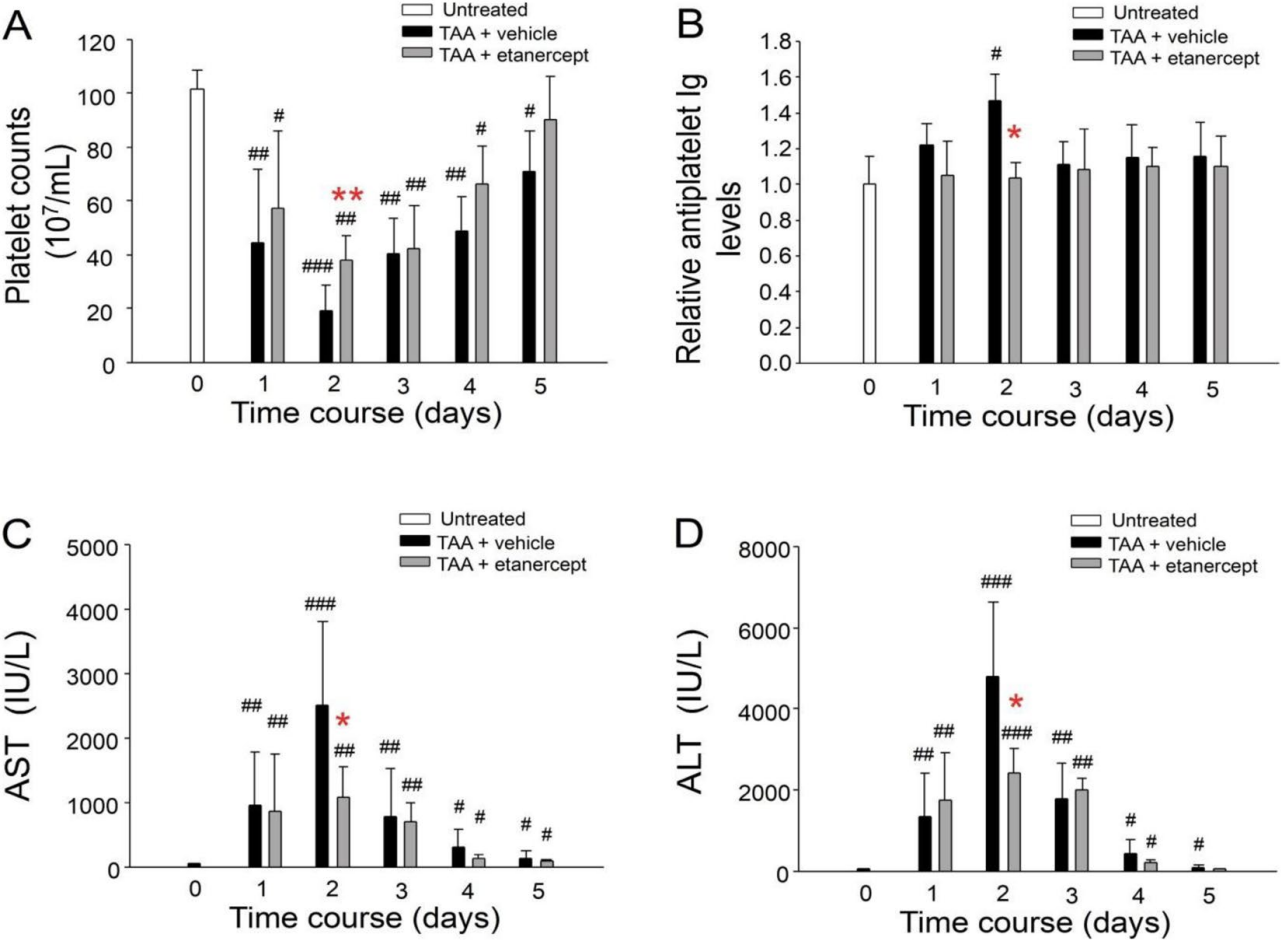
Anti-TNF treatments rescued TAA-induced liver injury and associated elevation of anti-PLT Ig and thrombocytopenia in mice. The platelet counts (**A**), circulating anti-PLT Ig (**B**; Day 0 groups were normalized to 1 fold), AST (**C**) and ALT (**D**) levels were analyzed in the TAA-mouse model with or without anti-TNF drug etanercept treatments. n = 6, ^#^*P* > 0.05, ^##^*P* > 0.01, ^###^*P* > 0.001, vs. respective day 0 groups (untreated); *P > 0.05; **P > 0.01 vs. respective TAA + vehicle groups.

**Figure 6:**
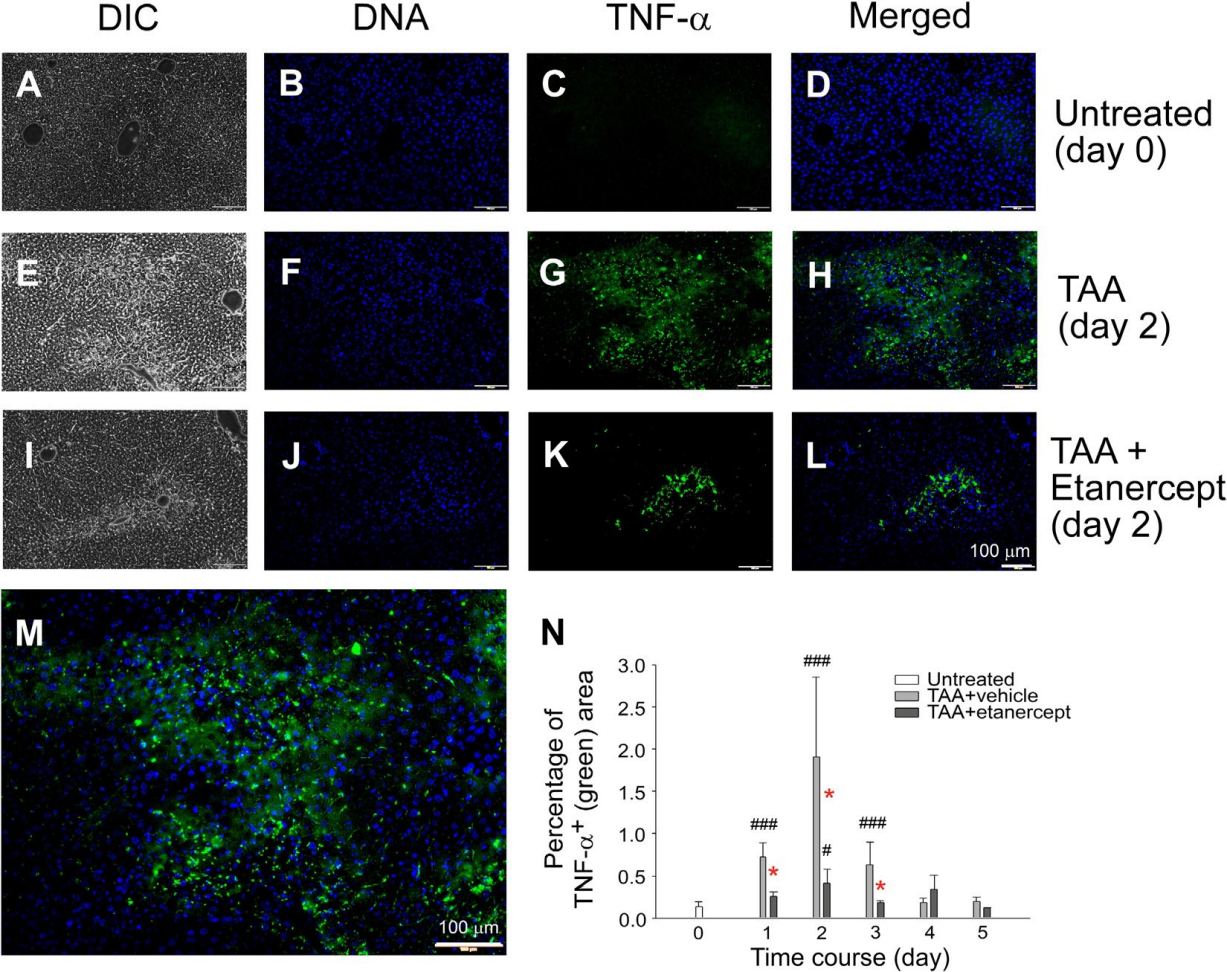
Anti-TNF treatments reduced TAA-induced TNF-α expression in mouse liver. The IHC results on liver TNF-α expression in TAA-challenged C57BL/6J mice with (**I**–**L**), or without (**E**–**H**) additional anti-TNF etanercept treatments was compared to untreated controls (**A**–**D**). Representative images are showed. To highlight the image details of TNF-α staining, M is showed as an enlarged image of H. DIC: bright field of differential interference contrast image; DNA: stained with DAPI. Image J quantified TNF-staining signals are showed in N. ^#^*P* < 0.05, ^###^*P* < 0.001 vs. day 0 groups; *P < 0.001 vs. respective vehicle groups. Quantified results were analyzed using at least 3 independent images in each group. Scale bars 100 μm.

Liver damage in TAA-treated mouse livers was observed at the macroscopic level before and after formalin fixation, particularly in the sample of wild-type mice (Fig. 3A–H). H&E staining (Fig. 3I–L) revealed that necrotic lesions (arrows indicate these areas) formed after TAA treatment in both wild-type and BCD mice (Fig. 3I,K vs. J,L, respectively), despite the BCD mice livers exhibiting less damage than the wild-type mice livers (Fig. 3I–L). To analyse the necrotic area of mouse liver, IHC analyses indicated that the TAA-treated wild-type groups but not the vehicle-treated wild-type or BCD groups displayed strong TNF-α expression (Fig. 4; WT TAA day 2 vs. WT TAA day 0 groups), which is consistent with the ELISA analyses of circulating TNF-α levels (Fig. 2E), and the quantified image results (Fig. 4R, ***P < 0.001, WT vs. BCD). In addition, 2 days after TAA-treatments, we observed increased expressions of proinflammatory cytokine HMGB1 and IL-6 in the the circulation (Fig. 2F,G; ***P < 0.001, TAA vs. vehicle; ^###^*P* < 0.001, day 2 vs. day 0) and the liver (Figs. S4 and S5, ^###^*P* < 0.001, day 2 vs. day 0) of mice. To further investigate whether TNF-α is crucial to TAA-mediated pathogenesis, the anti-TNF drug etanercept was used. Data revealed that etanercept treatment ameliorated liver damage, thrombocytopenia, and the induction of anti-PLT Ig (Fig. 5, *P < 0.05, **P < 0.01, day 2, etanercept vs. vehicle). The IHC analyses further revealed that the anti-TNF treatments not only rescue the excesive production of TNF-α (Fig. 6, *P < 0.05, day 2, etanercept vs. vehicle), but also reduced the liver HMGB1 and IL-6 expression levels in mice (Figs. S6 and S7, *P < 0.05, **P < 0.01, day 2, etanercept vs. vehicle). These results suggest that TNF-α-mediated inflammation is associated with TAA-induced pathogenic responses.

## Discussion

Thrombocytopenia has been demonstrated to be related to the degree of liver damage in both clinical cases and animal models^47–49^. Because the liver is the major organ expressing TPO, discovering thrombocytopenia in patients with chronic liver diseases would be expected^1–5^. However, whether thrombocytopenia and anti-PLT Ig are induced during the acute phase of liver diseases is unclear. Despite severe hepatic dysfunction, serum TPO level was normal and not associated with platelet count in acute liver failure in one study^50^, suggesting that additional pathological factors are more critical. Because coagulation pathways have been demonstrated to be activated during acute liver injury and antiplatelet therapy has decreased liver injury and mortality^48,49,51^, the activation of platelet-related coagulation pathways are likely to be part of the pathophysiology. As the mechanism remains elusive, various candidate pathogenic factors may be involved^5^. Circulating anti-PLT Ig has been shown to activate platelets and the coagulation system and has frequently been associated with liver diseases^9,52^. Platelet and anti-PLT Ig complexes have also been demonstrated to exacerbate excessive inflammation and coagulation^53,54^. This evidence collectively suggests that anti-PLT Ig is a candidate pathogenic factor that contributes to thrombocytopenia in acute liver diseases, although its pathological role remains to be investigated.

Using a mouse model, we discovered that TAA-induced acute liver damage is associated with the induction of thrombocytopenia and increased plasma anti-PLT Ig, and TNF-α levels (Figs. 1 and 2). These responses are partially consistent with the pathological changes observed in patients with recurrent hepatitis in the acute phase (Fig. 1A–C). By contrast, BCD mice, which lacked circulating Ig expression, exhibited markedly lower pathogenic responses after TAA treatment. Because no foreign antigens (e.g. hepatitis virus) were challenged, a conventional adaptive autoimmune response through the molecular mimicry of viral antigens was not involved; instead, such a pathogenic immune response was more likely due to excess-inflammation-induced aberrant B cell activation^43–46,55,56^. TNF-α is a pleiotropic cytokine and key mediator of inflammation that regulates numerous physiological functions and is an essential element in the development of autoimmunity^57–60^. Proinflammatory stimulation through interferon, TNF, and toll-like receptors has been demonstrated to stimulate B cell activation, proliferation, and differentiation^45,46,55,56,61,62^, in which the TNF could be either externally supplied^55^ or exerted as an autocrine growth factor^56^. The involvement of TNF-α has been identified in the pathogenesis of rheumatoid arthritis, and the blockade of TNF-α has proven to be an effective treatment for patients with rheumatoid arthritis and Crohn’s disease^57,60^. This evidence collectively suggests that excessive inflammation leads to aberrant B cell activation and Ig secretion^43–46,55,56^ and may explain why severe liver damage induces anti-PLT Ig and thrombocytopenia in wild-type mice but not BCD mice after TAA treatment (Fig. 2). Anti-TNF treatment markedly ameliorated the TAA-mediated induction of anti-PLT Ig, circulating AST and ALT levels, and thrombocytopenia in mice (Fig. 5). This suggests that a TNF-mediated inflammatory pathway contributes to the pathogenesis. Accordingly, we proposed a hypothetical model (Fig. S8). As anti-TNF treatments reduced anti-PLT Ig production (Fig. 5B), this suggested that the TAA-induced TNF production is prior to the elicitation of anti-PLT Ig (Fig. S8). In addition, B-cell deficient phenotype ameliorates TAA-induced liver damage in BCD mice, further suggests that antibodies, including the anti-PLT Ig fractions, may play some roles on the exacerbation of liver damages (Fig. S8). Circulating anti-PLT Ig was only observed in the acute phase of liver damage, and it diminished during convalescence (Fig. 1); thus the induction of anti-PLT Ig is a temporary but not long-lasting autoimmune response. Given that autoantibodies are induced in various liver diseases^63,64^, whether such an autoimmune-prone condition facilitates the development of long-term liver-related autoimmune disease is worthy of further investigation.

Despite revealing a short-term feature, our data suggest that liver-damage-induced anti-PLT Ig may partially exacerbate liver damage. The direct immunisation of platelets to elicit anti-PLT Ig can induce liver damage in mice (Fig. S9), indicating the negative role of anti-PLT Ig in liver injury. Anti-PLT Ig and platelet complexes have been shown to accumulate in the liver^65,66^. Platelet depletion before but not after liver injury ameliorates liver diseases, probably because of liver-accumulated platelet-elicited inflammation^48,67,68^. This is in agreement with our TAA-mouse model using BCD mice, in which TAA treatment induced only limited liver damage and thrombocytopenia in hosts lacking Ig expression (Fig. 2, BCD groups), suggesting a negative role of anti-PLT Ig in liver diseases.

In summary, we discovered that acute liver damage is associated with temporarily induced circulating anti-PLT Ig level and thrombocytopenia. BCD mice that lack functional B cells and Ig expression exhibited markedly less thrombocytopenia and liver damage, implying a negative role of the antibody in this TAA-mouse model. Anti-TNF treatment ameliorated both low platelet count and liver injury, revealing the involvement of the TNF pathway. Additional investigations are required to decipher the mechanism in detail and determine the pathological impact of anti-PLT Ig on acute liver diseases, and doing so may be helpful for the development of feasible therapeutic approaches against liver diseases and liver-damage-associated coagulation defects.

## Supplementary information

Supplementary Information
