# Supplementary material for: Thioacetamide-induced liver damage and thrombocytopenia is associated with induction of antiplatelet autoantibody in mice
Source: Sci Rep. 2019 Nov 25;9:17497. doi: 10.1038/s41598-019-53977-7 (PMC6877565; doi:10.1038/s41598-019-53977-7)
Supplement: Supplementary file 1 — Supplementary Information [file 41598_2019_53977_MOESM1_ESM.pdf]

Supplementary Information:

Thioacetamide-induced liver damage and thrombocytopenia is associated with induction of antiplatelet autoantibody in mice

You-Yen Lin[1†], Chi-Tan Hu[2, 3, 4†], Der-Shan Sun[1,3], Te-Sheng Lien[3] and Hsin-Hou Chang[1,3]

[1] Institute of Medical Science, Tzu-Chi University, Hualien, Taiwan, R.O.C.
[2] Research Center for Hepatology, and Department of Gastroenterology, Buddhist Tzu Chi General Hospital, Hualien, Taiwan, R.O.C.
[3] Department of Molecular Biology and Human Genetics, Tzu-Chi University, Hualien, Taiwan, R.O.C.
[4] School of Medicine, Tzu-Chi University, Hualien, Taiwan, R.O.C.

†Share equals contribution.
Correspondence should be sent to H.-H.C.
Institute of Molecular and Cellular Biology, Tzu-Chi University,
Room D407, No. 701, Chung Yang Rd., Sec. 3, Hualien 970, Taiwan, R.O.C.
Tel: 886-3-8565301 ext 7296. Fax: 886-3-8578386.
E-mail:hhchang@mail.tcu.edu.tw

Supplementary Figures. S1-S9

Supplementary Fig. S1.

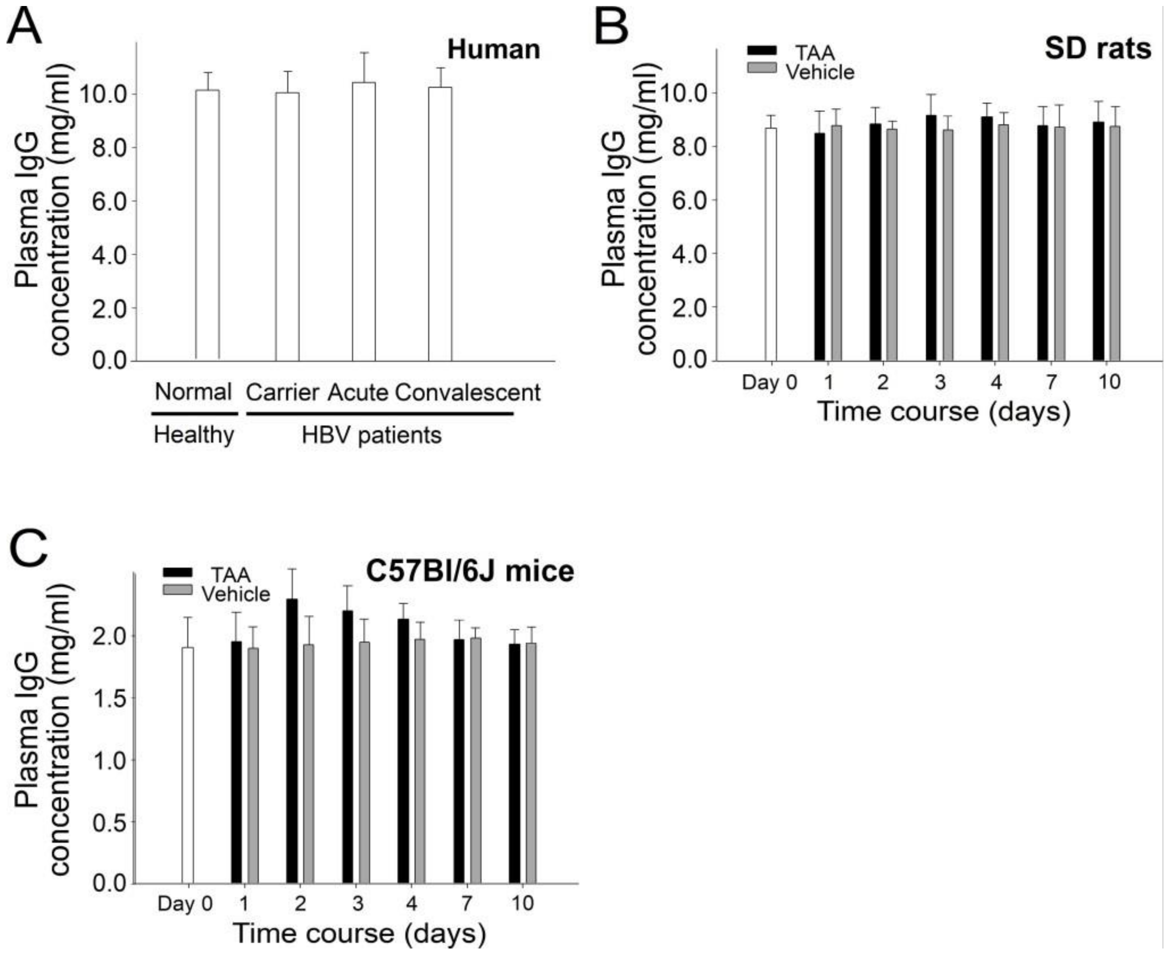

Supplementary Fig. S1. Total IgG levels. The plasma total IgG levels in human subjects, rats and mice were determined. Liver damages tended to enhance the circulating IgG levels in mice. Normal healthy control n = 6, HBV patients n = 5 (A); n = 18 (B); n = 6 (C).

Supplementary Fig. S2.

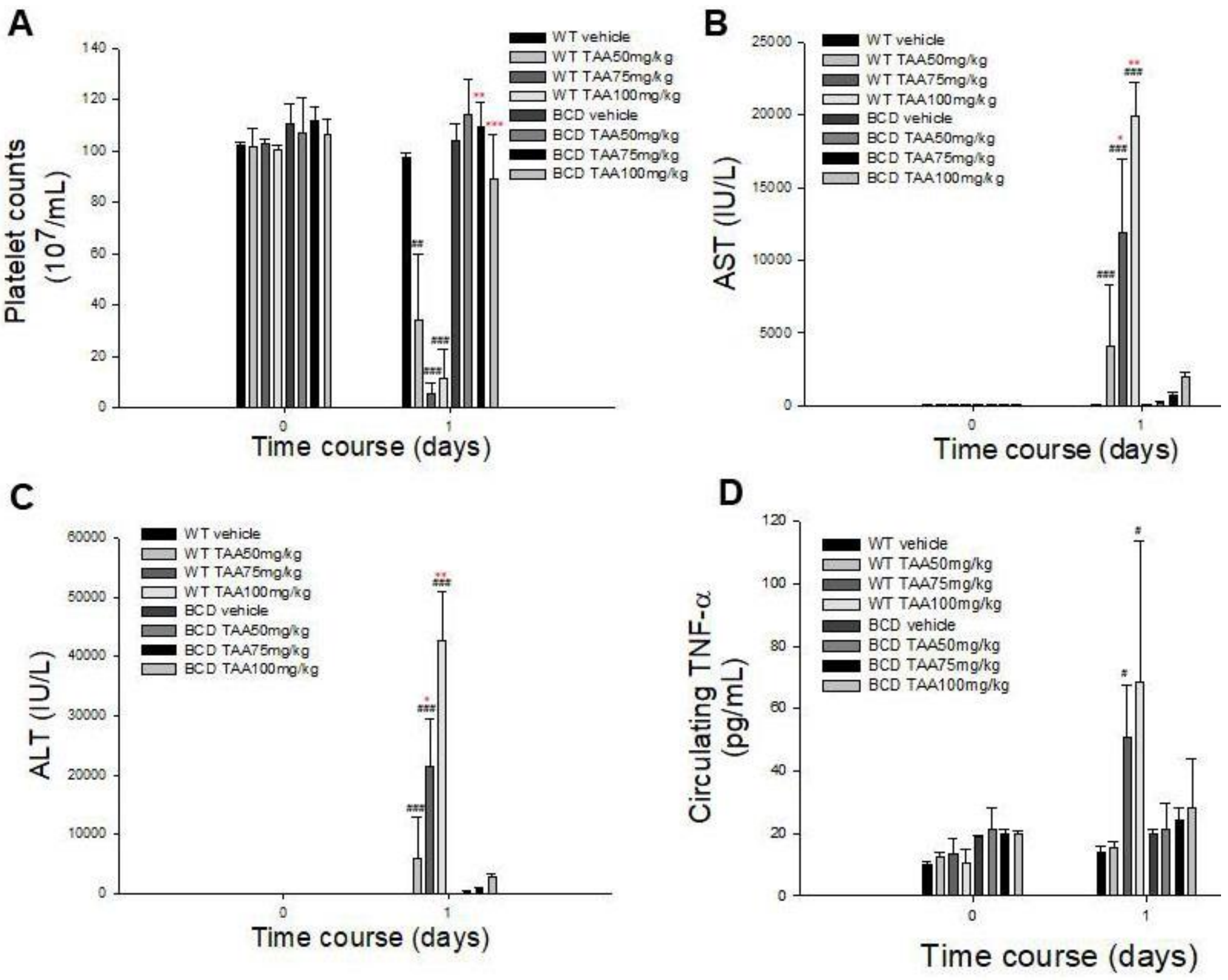

Supplementary Fig. S2. TAA treatments induced thrombocytopenia, liver damage and inflammation in mice. Wild type (WT) and B cell deficient (BCD) mice were treated with (50 mg/kg, 75 mg/kg and 100 mg/kg) or without TAA. The levels of their circulating platelet counts, liver enzymes and TNF were analyzed. We found that TAA treatments induced thrombocytopenia, liver damage and inflammation in WT mice in a dose dependent manner, and to a less extend in BCD mice. # $P < 0.05$, ## $P < 0.01$, ### $P < 0.001$ vs. respective day 0 groups; * $P < 0.05$, ** $P < 0.01$, *** $P < 0.001$ WT vs. BCD groups. n = 6.

Supplementary Fig. S3.

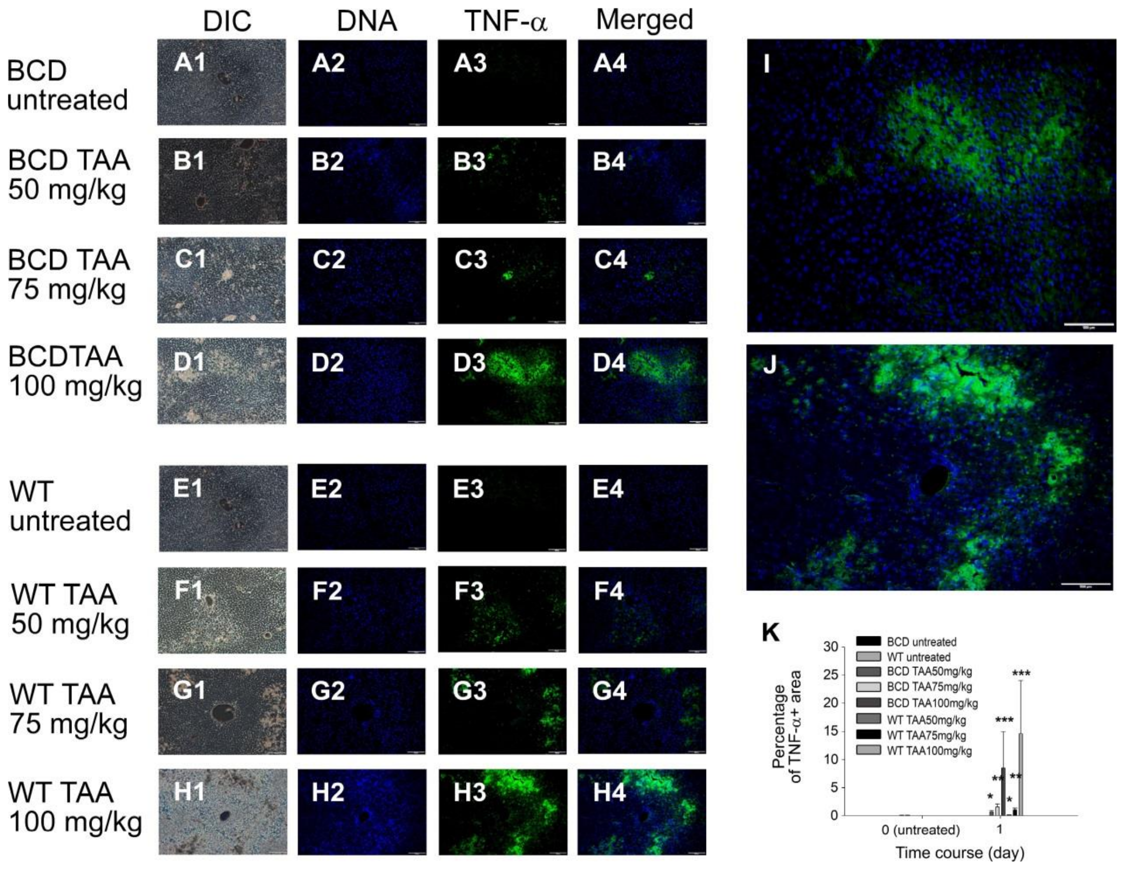

Supplementary Fig. S3. TAA treatments induced high level TNF-α expression in the mouse liver. Representative immunohistochemistry (IHC) images (A-J) and the quantified results (K) are showed. Wild type (WT) mice displayed much higher TNF expression levels in the liver after TAA treatments, than that of B cell deficient (BCD) mice. To illustrate the image details of TNF-α staining, I and J are showed as enlarged images of D4 and H4, respectively. DIC: bright field of differential interference contrast image; DNA: stained with DAPI. * $P < 0.05$, ** $P < 0.01$, *** $P < 0.001$ vs. respective day 0 groups. Scale bars 100 μm. Because the TAA dosage higher than 75 mg/kg (iv) cause high mortality and high variation in mouse, we conducted following experiments using the dosage 50 mg/kg.

Supplementary Fig. S4.

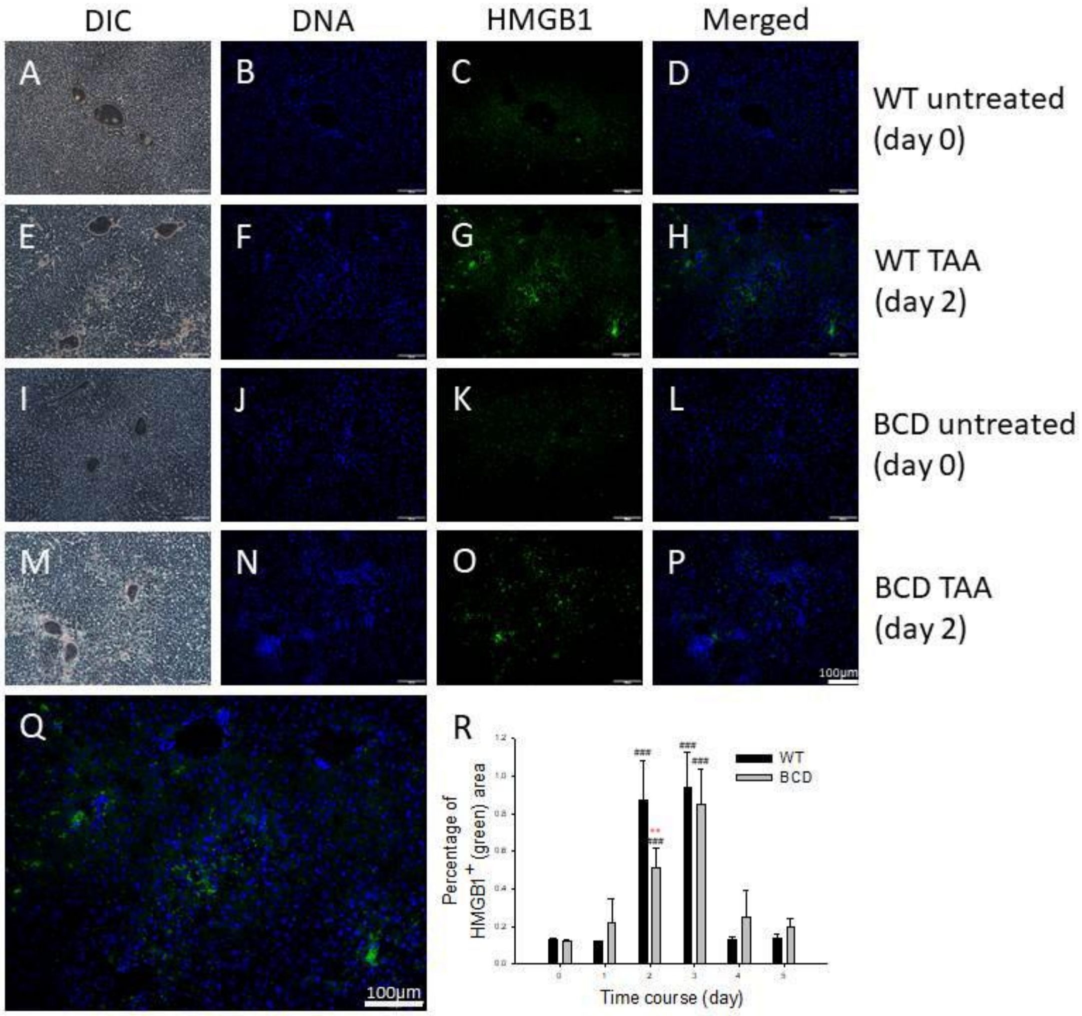

Supplementary Fig. S4. TAA treatments induced high level HMGB1 expression in the mouse liver. Wild type (WT) and B cell deficient (BCD) were analyzed. Representative immunohistochemistry (IHC) images (A-Q) and the quantified results (R) are showed. To illustrate the image details of cytokine staining, Q is showed as an enlarged image of H. DIC: bright field differential interference contrast image; DNA: stained with DAPI. ### $P < 0.001$ vs. respective day 0 groups; ** $P < 0.01$ vs. respective WT groups. Quantified results were analyzed using at least 3 independent images in each group. Scale bars 100 μm.

Supplementary Fig. S5.

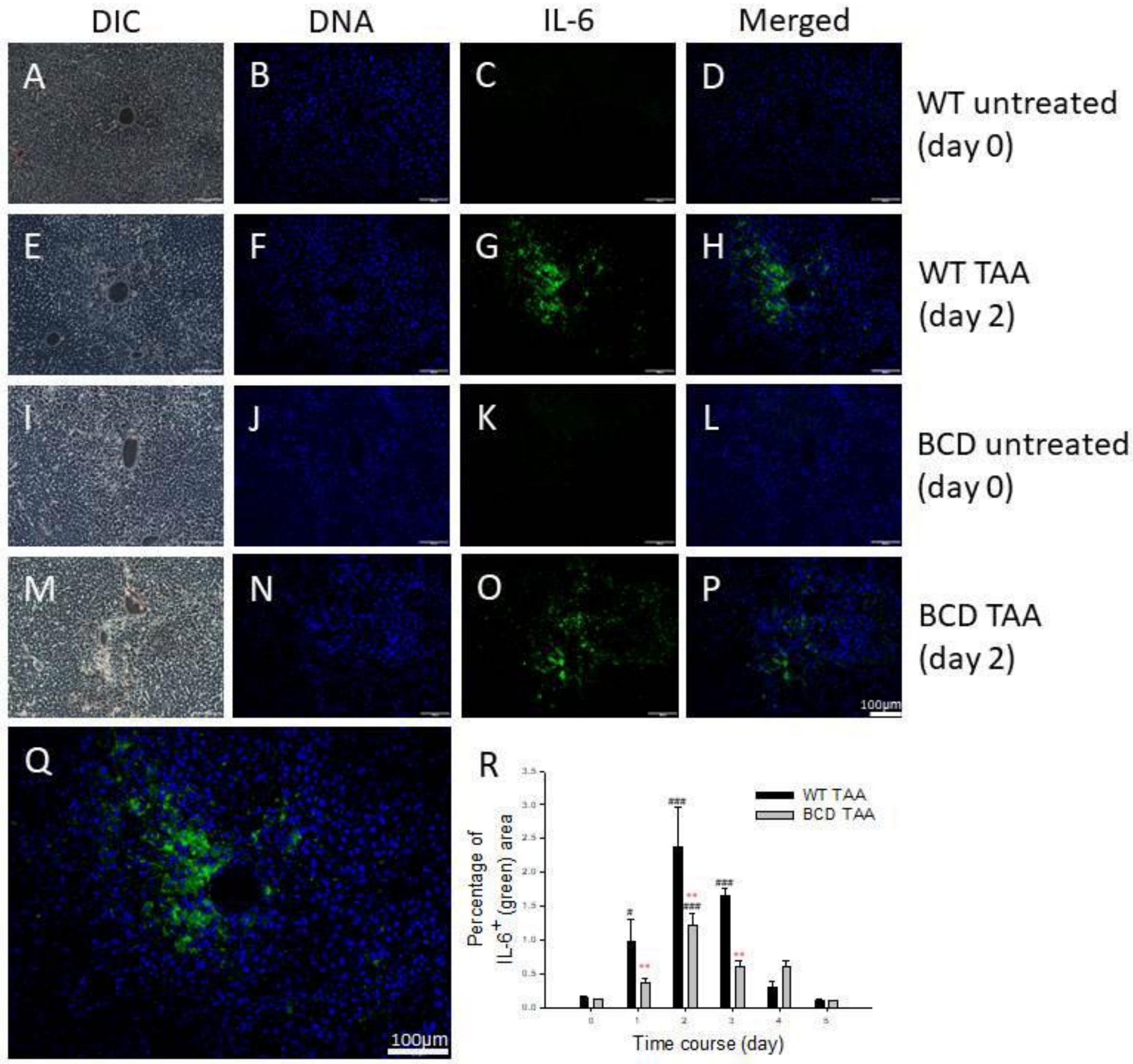

Supplementary Fig. S5. TAA treatments induced high level IL-6 expression in the mouse liver. Wild type (WT) and B cell deficient (BCD) were analyzed. Representative immunohistochemistry (IHC) images (A-Q) and the quantified results (R) are showed. To illustrate the image details of cytokine staining, Q is showed as an enlarged image of H. DIC: bright field differential interference contrast image; DNA: stained with DAPI. # $P < 0.05$, ### $P < 0.001$ vs. respective day 0 groups; ** $P < 0.01$ vs. respective WT groups. Quantified results were analyzed using at least 3 independent images in each group. Scale bars 100 μm.

Supplementary Fig. S6.

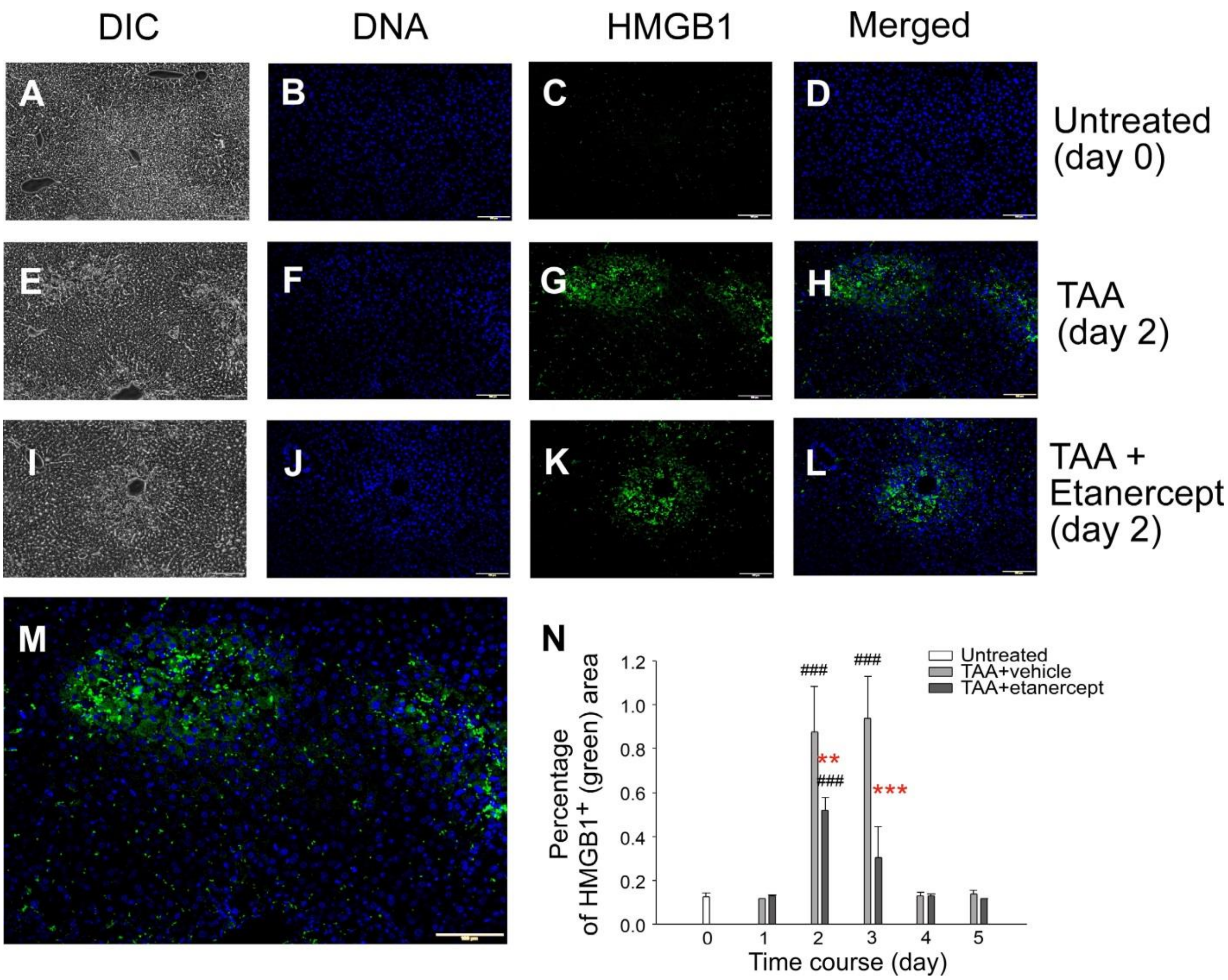

Supplementary Fig. S6. Anti-TNF treatments rescued TAA treatment-induced high level proinflammatory cytokine HMGB1 expression in the mouse liver. TAA-treated mice with or without anti-TNF etanercept treatments were analyzed. Representative immunohistochemistry (IHC) images (A-M) and the quantified results (N) are showed. To illustrate the image details of cytokine staining, M is showed as an enlarged image of H. DIC: differential interference contrast bright field image; DNA: stained with DAPI. ### $P < 0.001$ vs. respective day 0 groups; ** $P < 0.01$, *** $P < 0.001$ vs. respective vehicle groups. Quantified results were analyzed using at least 3 independent images in each group. Scale bars 100 μm.

Supplementary Fig. S7.

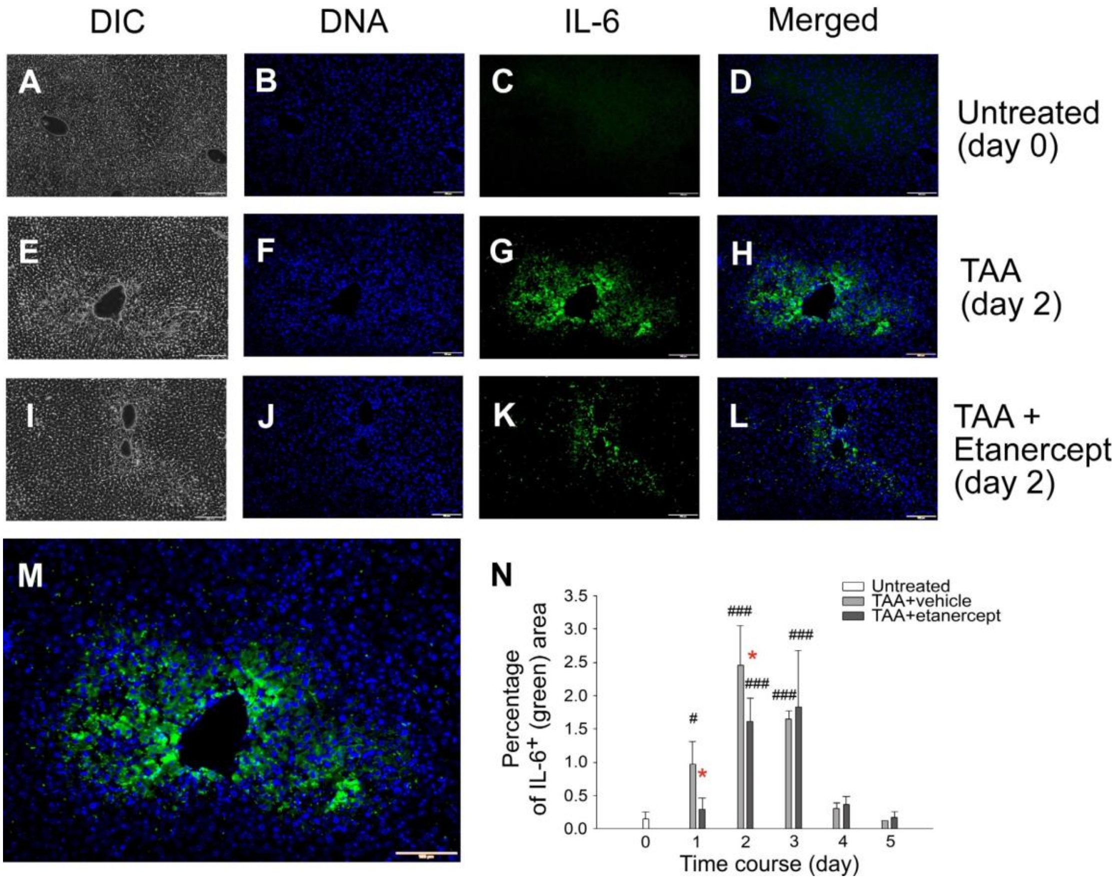

Supplementary Fig. S7. Anti-TNF treatments rescued TAA treatment-induced high level proinflammatory cytokine IL-6 expression in the mouse liver. TAA-treated mice with or without anti-TNF etanercept treatments were analyzed. Representative immunohistochemistry (IHC) images (A-M) and the quantified results (N) are showed. To illustrate the image details of cytokine staining, M is showed as an enlarged image of H. DIC: differential interference contrast bright field image; DNA: stained with DAPI. ### $P < 0.001$ vs. respective day 0 groups; ** $P < 0.01$, *** $P < 0.001$ vs. respective vehicle groups. Quantified results were analyzed using at least 3 independent images in each group. Scale bars 100 μm.

Supplementary Fig. S8.

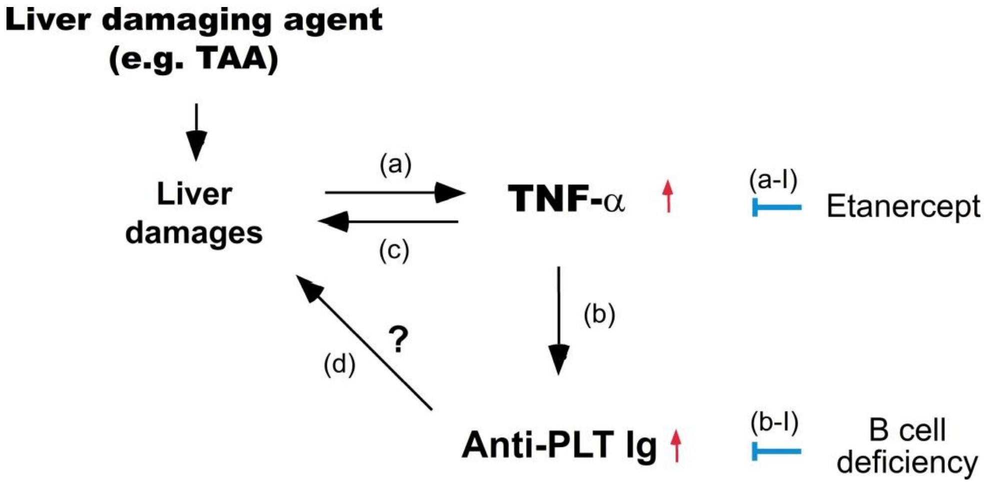

Supplementary Fig. S8. A hypothetical model. According to the analysis results, a hypothetical model is proposed. Treatments of TAA will induce liver damage and TNF-α production (a). The elevation of TNF-α is likely involving in the stimulation of antiplatelet autoantibody (anti-PLT Ig) production (b) and subsequently TNF-α mediated liver damages (c), because anti-TNF etanercept treatments can ameliorate TAA-induced elevation of anti-PLT Ig, AST and ALT levels (a-I) (Fig. 5). In addition, B cell deficient mice (BCD) with IgG expression (including anti-PLT Ig) deficiency, displayed a less liver damage during TAA treatments (b-I) (Fig. 2), suggesting a role of anti-PLT Ig on the stimulation of further liver damage (d), while the detailed mechanism remains to be further investigated. Black arrows: induction or enhancement; red small arrows: increased levels.

Supplementary Fig. S9.

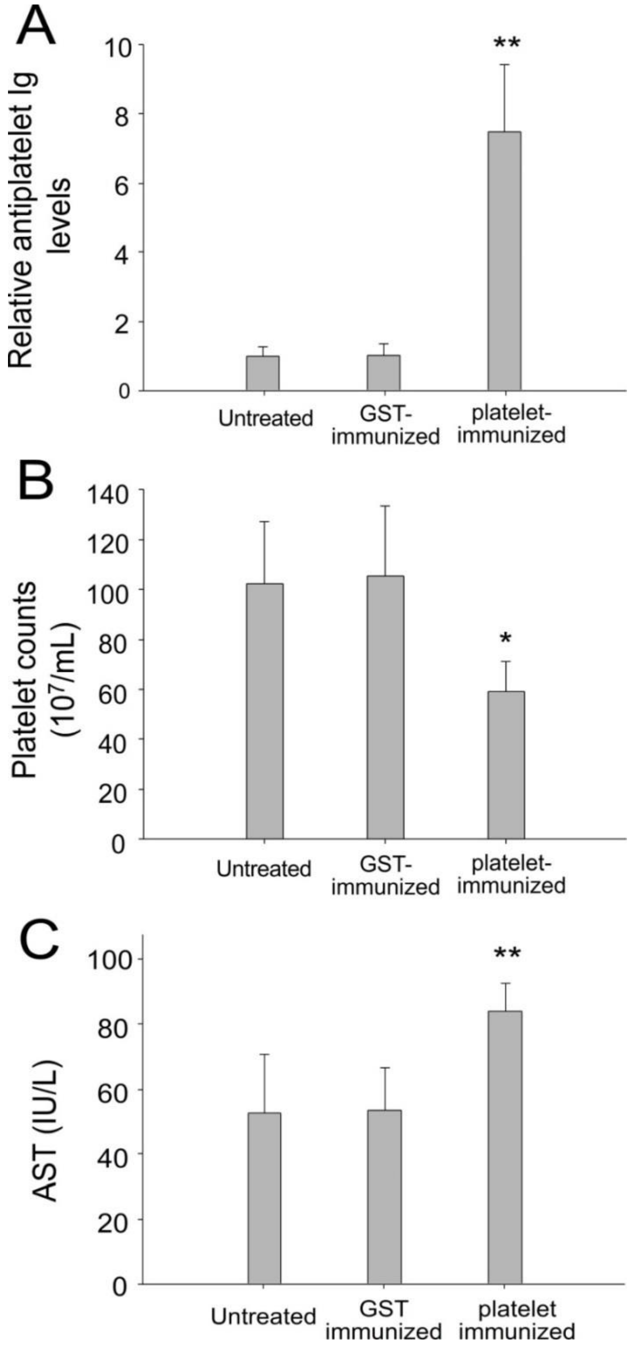

Supplementary Fig. S9. Immunization of rabbit platelets induced anti-PLT Ig, low platelet counts and liver damage in mice. The circulating anti-PLT Ig (A), platelet counts (B) and AST (C) levels were analyzed after mice were immunized with purified rabbit platelets. n = 6, * $P > 0.05$; ** $P > 0.01$ vs. respective untreated (without immunization) groups.
